# An expansile presentation of focal cemento-osseous dysplasia of the mandible in a young girl

**DOI:** 10.1259/bjrcr.20230013

**Published:** 2023-05-11

**Authors:** Jonas Ver Berne, Reinhilde Jacobs, Esther Hauben, Constantinus Politis

**Affiliations:** 1 Department of Oral and Maxillofacial Surgery, University Hospitals Leuven, Leuven, Belgium; 2 OMFS-IMPATH Research Group, Department of Imaging and Pathology, Catholic University, Leuven, Belgium; 3 Department of Dentistry, Karolinska Institutet, Solna, Sweden; 4 Department of Pathology, University Hospitals Leuven, Leuven, Belgium

## Abstract

A 6-year-old girl presented with a grossly expansive lesion of the left lower jaw. Radiological investigations revealed a large mixed radiolucent/radio-opaque lesion of the left mandible extending into the ramus. Correlation of biopsy and imaging results lead to the diagnosis of an expansile form of focal cemento-osseous dysplasia. Surgical enucleation was performed, and the patient remained free of recurrence after 6 months of follow-up. When dealing with fibro-osseous lesions of the jaw, correlation of radiological and pathological results is mandatory to make a correct diagnosis and avoid unnecessarily extensive surgery.

## Case presentation

A 6-year-old girl of North-African origin was referred to the maxillofacial surgery clinic with an expansive mass in the left lower jaw. Clinically, she exhibited a marked swelling of the left lower jaw without pain, neurosensory disturbances, or functional impairment. On intraoral examination, there was expansion of the buccal aspect of the left mandible with normal inspection of the mixed dentition.

## Radiological investigations

**Figure 1.**
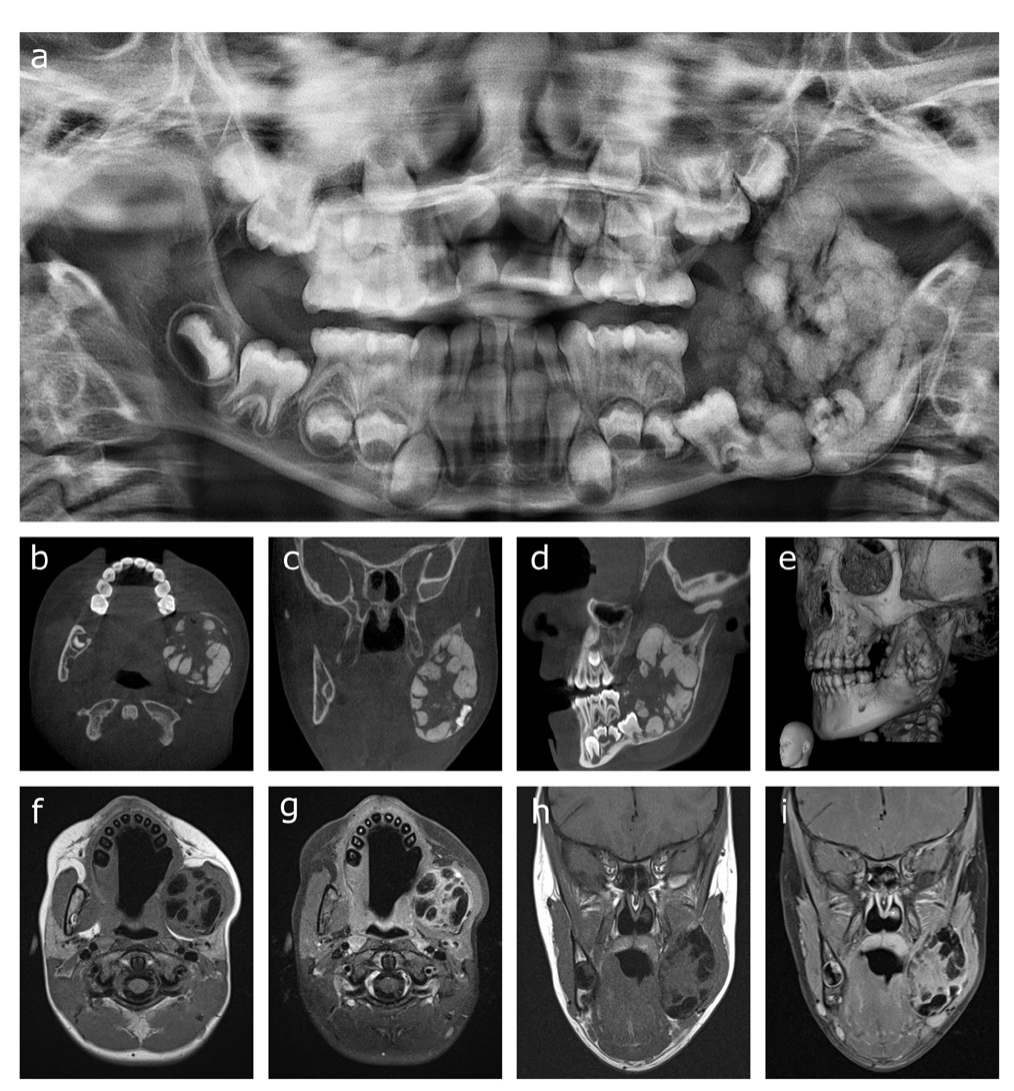
(**a–i**) Panoramic imaging of the expansive lesion in the left mandibular ramus showing a cloudy appearance of the opacities inside the lesion, expansion and erosion of the inferior cortex, and the thin radiolucent line separating it from the internal lobular masses (**a**). Cone beam CT showing the lesion containing multiple ginger root-like lobules, impacted first and second left mandibular molars, and showing expansion and erosion of the inferior cortex with a thin radiolucent line separating it from the lesion (**b–e**). MRI showing T1 hypointense lobular masses on a monotonous isointense background (**f, h**). T1-fat suppressed images showing attenuation of the background tissue after gadolinium injection (**g, i**).

Panoramic imaging of the jaws showed the mixed radiolucent/radio-opaque lesion in the left mandible with cloudy internal opacities and expansion and erosion of the inferior mandibular cortex (Figure 1a). A thin radiolucent rim was observed separating the lesion from the inferior mandibular cortex. Cone beam CT (CBCT) showed the voluminous mass in the left ramus of the mandible, reaching from the second mandibular deciduous molar up to the coronoid process. There was erosion of the medial and lateral cortices with focal interruption of its delineation (Figure 1b–e). Unerupted first and second left mandibular molars were contained in the lesion along with numerous plump calcifications with ginger root-like appearance. Additional MRI was performed for further characterization of the lesion and evaluation of soft tissue extension (Figure 1f–i). The center of the lesion appeared heterogeneous with multiple hypointense components on a T1- and T2-isointense background. After gadolinium contrast injection, there was attenuation of the center of the lesion except for the hypointense components. Invasion into neighboring structures was not observed and there were no enlarged lymph nodes.

### Radiological differential diagnosis

**Figure 2.**
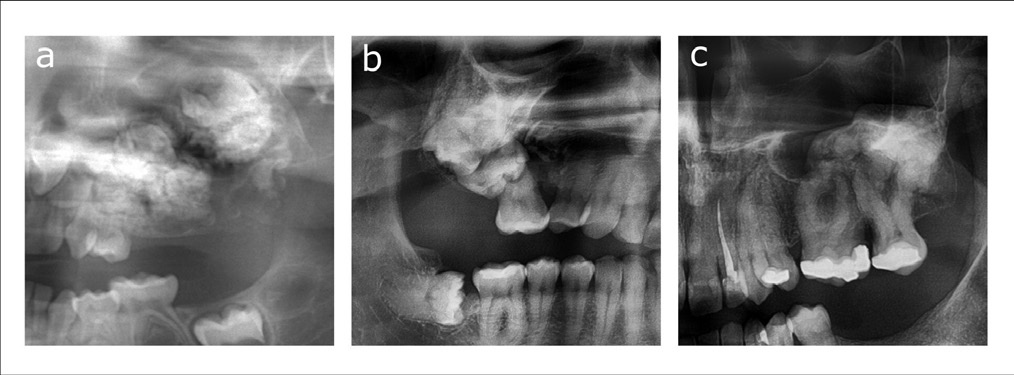
(**a–**c) Panoramic images showing the differential diagnoses for the present case: ameloblastic fibro-odontoma appearing as an expansive lesion with internal cloudy appearance of the left maxilla (**a**), complex odontoma around an impacted second right maxillary molar (**b**), and cemento-ossifying fibroma of the left posterior maxilla expanding into the maxillary sinus (**c**).

The main radiological differential diagnoses for this lesion were the groups of odontogenic tumors (ameloblastic fibro-odontoma/fibrodentinoma and complex odontoma) and fibro-osseous lesions [cemento-ossifying fibroma and expansive focal cemento-osseous dysplasia, (COD)]. An ameloblastic fibro-odontoma/fibrodentinoma was listed in the WHO classification of Head and Neck Tumors 2005, 3ed edition as an ameloblastic fibroma forming enamel/dentine, giving the typical radiographical appearance of a mixed radiolucent/radio-opaque lesion. It was considered a neoplastic lesion displaying significant bony expansion. The internal radio-opacities ranged from a fine speckled appearance to large lobular calcifications (Figure 2a). In the WHO Classification of Head and Neck Tumors 2017, 4th edition this entity was no longer recognized and is now considered a developing odontoma containing a prominent soft tissue part.^1^ However, clinical and radiographical features still suggest that at least a subset of these ameloblastic fibro-odontoma/fibrodentinomas behave differently from odontomas. Their appearance in younger patients (cut-off age of 13.5 years) than do odontomas and their larger size (cut-off size of 2.1 cm) are conflicting with the notion that they are *developing* odontomas.^2^ Moreover, in ameloblastic fibro-odontoma and ameloblastic fibro-dentinoma the BRAF p.V600E mutation is detected in respectively 34 and 60% of the cases, suggesting that some of them are related to ameloblastic fibroma.^3^ In the upcoming 2022 WHO classification of Head and Neck Tumors, the ameloblastic fibro-odontoma is still excluded as a separate entitiy awaiting further elucidation by molecular study.^4^

Odontomas are listed in the 2017 WHO classification of Head and Neck Tumors as benign mixed epithelial–mesenchymal odontogenic tumors. As a subset, complex odontomas are a haphazard assembly of enamel, dentin, cementum, and dental pulp tissue and appear on radiographs as mixed radiolucent/radio-opaque lesions. The degree of radio-opacity can vary greatly between lesions depending on the degree of development, but a characteristic radiolucent rim around the lesion is observed. Odontomas are commonly associated with impacted teeth, and large lesions can cause expansion of the cortices (Figure 2b). In the impending 2022 WHO classification of Head and Neck Tumors, odontomas are considered hamartomatous lesions of developing teeth rather than neoplasms, since they stop growing when the dentition is fully developed.^4^ This means that extreme sized lesions are rare.

Cemento-ossifying fibroma is a benign mesenchymal odontogenic neoplasm while also belonging to the group of fibro-osseous lesions. It is an expansive lesion showing erosion of the cortex and contains variable amounts of internal opacification, ranging from completely radiolucent to mixed to completely radio-opaque (Figure 2c). As opposed to the juvenile variants, cemento-ossifying fibromas show a thin radiolucent rim separating it from the surrounding bone, although this is difficult to appreciate when lesions are predominantly radiolucent.

COD, also a fibro-osseous lesion, can be divided into focal and florid types, familial and non-familial types, and an expansive type. The expansive COD is a rare entity presenting as a single expanding lesion in a non-familial capacity. On radiographic imaging, all the features of focal or periapical COD are present: a mixed radiolucent/radio-opaque lesion with ginger root-like opacities and a radiolucent rim, but with extension into the basal bone in contrast to the other types which only appear in the alveolar parts of the jaws.^5^ A classic maturation pattern is observed in these dysplastic lesions, starting with a radiolucent lesion that exhibits progressive opacification.

### Histopathological differential diagnosis

An intraoral biopsy was performed which showed a variable cell-rich, non-circumscribed, non-encapsulated growing spindle cell collagenous tumoral proliferation, both morphologic and after extensive immunohistochemical analysis without obvious bone/chondroid matrix formation or (odontogenic) epithelial elements. The morphology resembled desmoid-type fibromatosis of the soft tissues, making the appearance compatible with a desmoplastic fibroma of bone. Additional DNA-RNA Next Genome Sequencing did not reveal any mutations or gene rearrangements and FISH for MDM2 was negative, excluding any BRAF-related odontogenic lesions, fibrous dysplasia, and low-grade osteosarcoma. The lesion was negative for EMA, MUC-4, Prekeratine (AE1/AE3), and S100. Considering the aforementioned morphological and extensive additional immunohistochemical examination, the diagnosis of a desmoplastic fibroma of bone was proposed.

Radiographically, however, desmoplastic fibroma of the mandible presents as a multilocular radiolucent lesion with sharp cortical borders and course straight septae, which was not the case in this patient. Expansion of the buccal and lingual cortices is seen in large lesions and even in small lesions cortical erosion is observed reflecting its infiltrative nature. Thus, even with the pathology report matching the desmoplastic fibroma, the lobular radio-opacities described on both panoramic and CBCT imaging made the pathological result incompatible with radiological findings in our patient.

### Treatment and final diagnosis

**Figure 3.**
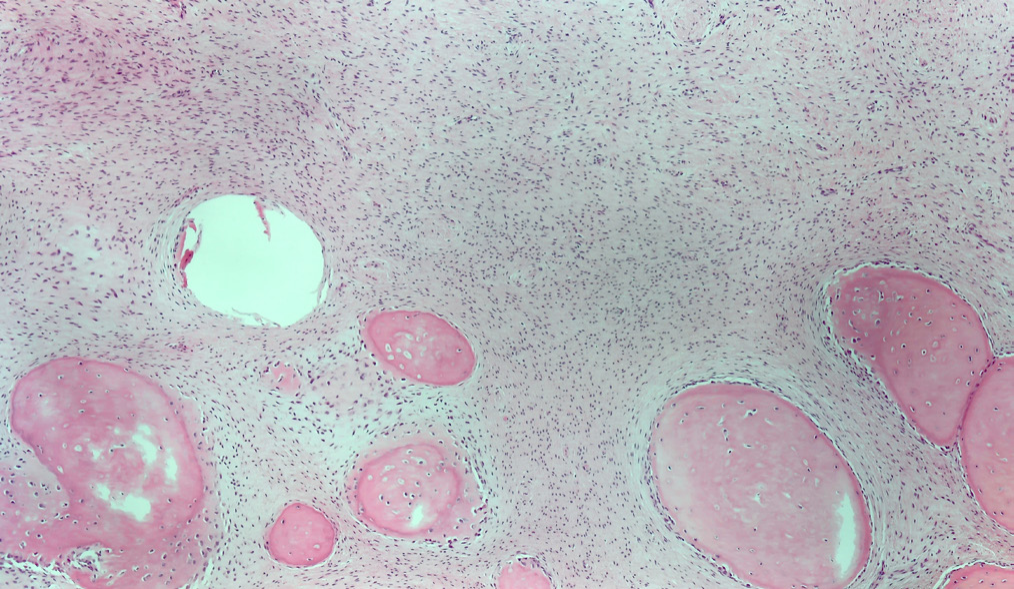
Photomicrograph illustrating the appearance of the resected lesion. In the upper half the monotonous, collagenous spindle cell component, which forms the bulk of the lesion and was the only component seen in the initial intraoral biopsy. In the lower half, the particles of woven bone(/cementum), with osteoblast(/cementoblast) rimming.

Correlating the fibrous morphology on biopsy and the lobular osseous/cemental masses on imaging, a working diagnosis of a fibro-osseous lesion was made, and surgical enucleation performed. During surgery, the lesion was easily pealed from the surrounding mandibular bone, and the specimen consisted of many lobular bony masses. Final pathological examination showed the same monotone cell-poor stroma as present in a desmoplastic fibroma of bone. However, the lesion contained multiple particles of woven bone that appeared to be formed from the stroma itself (Figure 3). Although the histological appearance was not typical at first, the findings are compatible with a fibro-osseous lesions. After radiological correlation, the final diagnosis of an expansile case of focal COD was made.

**Figure 4.**
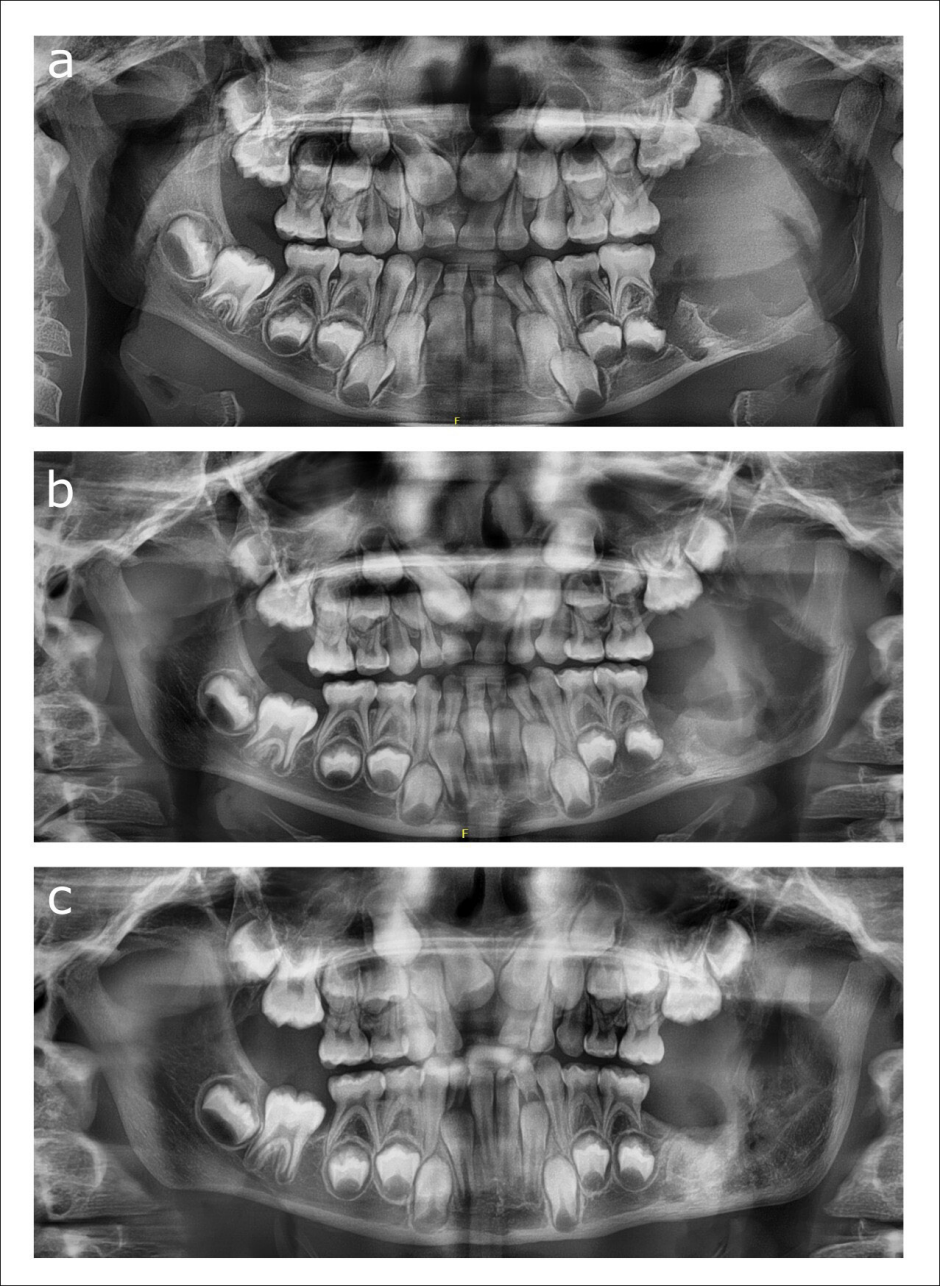
(**a–c**) Panoramic radiographs showing the postoperative course after enucleation of the lesion: 1 week after surgery showing the subcondylar fracture (**a**), 6 weeks after surgery (**b**), and 6 months after surgery showing progressive ossification of the left ramus (**d**).

A post-operative subcondylar fracture occurred on the left side given the thin rim of cortical bone that remained after enucleation, which was treated conservatively. After 6 months, progressive ossification of the defect was observed, and the patient exhibited no complaints (Figure 4a–c).

## Discussion

COD is a group of dysplastic lesions of presumably odontogenic nature. Periapical COD can be found at the apices of anterior mandibular teeth and, when solitary, often cause a differential diagnostic problem with periapical inflammatory conditions in its radiolucent stage. The finding of normal tooth vitality should eliminate the diagnosis of an inflammatory lesion. Focal COD has the same radiological appearance as periapical COD but presents as a solitary lesion in the premolar region. Florid COD involves the alveolar bone of multiple quadrants, sometimes causing minimal expansion of the cortices. Finally, an expansive type of COD, like the present case, has been described in the literature. Although the possibly enormous size of these lesions would justify the term *gigantiform*, Noffke et al proposed the better descriptor *expansive* COD. However, since most of the mineralized tissues in this lesion are not attached to the root surfaces, use of the term *cementum* or *gigantiform cementoma* to describe them should be discouraged.^6^ In contrast to the conventional types of COD, extension of the lesion into basilar bone is observed and occurs as a result of its grossly expansive nature.

The largest series of expansive COD has been reported by Raubenheimer et al who described 18 cases of non-familial lesions.^7^ Most patients were middle-aged, with one excess of 6-years-old. All cases showed gross expansion of the affected jaw, and most were in the anterior mandible. The most common associated pathologies in this series were conventional florid COD without expansion, tooth displacement, and simple bone cysts. Radiographically, most cases were unifocal. A radiolucent rim was observed around the periphery of dens mineralized lobules and cortical erosion was present. In expansive COD, the radiolucent areas in the center of the lesion correspond to the fibrous parts where expansion of the lesion occurs. With full opacification, these lesions stabilize, thus not exhibiting continuous growth like a neoplasm. The central radiolucent part should, however, not be confused with a simple bone cyst, which is often associated with fibro-osseous lesions.^6^ The diagnosis of an associated simple bone cyst should only be considered when its characteristic features are present (scalloping lesion between roots, minimal expansion) and the lesion is located adjacent to the fibro-osseous lesion, not within.

Fibro-osseous lesions of the jaw bones often have a characteristic appearance, although in some cases the differential diagnosis can be difficult. While panoramic imaging suffices for small lesions, additional imaging with CBCT is needed in large lesions for its capability of showing fine details otherwise difficult to appreciate. As depicted in Figure 2, the listed differential diagnoses in our case all have similar appearances on panoramic imaging. The imaging features of fibro-osseous lesions have been extensively reviewed by MacDonald.^8,9^ Pathological examination alone has not much contribution to the diagnosis of a fibro-osseus lesion, other than confirming that it is a fibro-osseus lesion, with the exception for entities characterized by specific molecular alterations (GNAS mutations for fibrous dysplasia and MDM2 amplification for some low-grade osteosarcomas). Clinical information including age, gender, race of the patient and radiological appearance of these lesions is invaluable and, in most cases, more contributive than histology to the exact diagnosis.

The non-expansive types of COD require no treatment. Even biopsies are contraindicated in these cases given the dense calcifications and lack of vascularization predisposing these lesions to massive infection. Only florid COD and expansive COD are surgically treated when they cause mass-effect and disfigurement of the facial appearance. Because of these differences in treatment, making a correct radiological and pathological diagnosis of fibro-osseous lesions is of utmost importance, as evidenced by the present case.

## Learning points

1. Expansive COD has a characteristic appearance with ginger root-like opacities on radiographic imaging. CBCT is a valuable addition to panoramic imaging in large lesions.
2. In small lesions, potentially expansive forms of COD can be recognized by (1) the young age of the patients, and (2) the greater volume of the radiolucent zones compared to conventional COD.
3. Careful radiological evaluation of mixed radiolucent/radio-opaque lesions is warranted. Often characteristic features can be identified even in similar appearing lesions.
4. When dealing with fibro-osseous lesions of the jaw, a final diagnosis can only be made when considering both pathological and radiological information. A precise diagnosis is beneficial for the patient, possibly avoiding resective surgery and subsequent reconstruction.
